# New Appliances and Things Medical

**Published:** 1895-06-08

**Authors:** 


### NEW APPLIANCES AND THINGS MEDICAL.

[We shall be glad to receive, at our Office, 428, Strand, London, W.C., from the manufacturers, specimens of all new preparations and appliances which may be brought out from time to time.]

### MITCHELL'S CASTOR OIL.

We have received from the British Castor Oil Company, 47, Victoria Street, S.W., a sample of the above castor oil. That most useful, but at the same time most generally disliked of purgatives, has long been considered the safest remedy of its class; the chief objection to it being its taste. We have never yet met with an oil that had not this taste till we tried the one mentioned above. Another objection is that when the oil is old, or expressed from old beans, it acquires not only a rancid acrid taste, but is distinctly irritant in its action, producing sometimes vomiting and diarrhœa. Now these objections are all of them ultimately due to the method of manufacture. "Cold drawn" castor oil may be expressed without heat, but it is exposed to air and moisture, hence fermentative changes, and consequently acrid action and vile flavour. The Mitchell process obviates all these difficulties. It expresses the oil in the cold at a constant temperature, and then filters it in vacuo, so that the absence of air and heat yields an oil which is bland, nutty in flavour, and of about twice the usual strength. The advantages in consequence in practice are the small dose and the absence of nausea produced by the filthiness of the medicine. We have tested the sample sent to us, both clinically and chemically, and the results are excellent. We find that this oil, too, makes an excellent mixture with glycerine, lemon juice, and oil of lemon—a severe test, for, when an ordinary sample is used, the taste of the castor oil always comes out, notwithstanding its covering of flavours. In conclusion, we consider this oil to be the best we have met with, and can cordially recommend its use to the profession.

### THE STANDARD MALT EXTRACT AND COD LIVER OIL.

(The Standard Malt Extract Company, 23, Billiter Street, E C.)

This firm has sent us for examination sample bottles of their Standard Malt Extract, with and without admixture with Cod Liver Oil. As is well known the value of a malt extract depends largely on the amount of diastase such a preparation contains, and we have requested our analyst to specially report on this point, as from our experience many of the malt extracts in the market are very deficient in diastasic value. The sample of malt extract examined contained 67·7 per cent. of maltose and 2·9 per cent. of carbohydrate calculated as dextrin. The diastasic value was determined by Lintner's process with soluble starch and Fehling's solution, and taking the diastasic value of a solution of 25 grams of malt in 500 c.c. of water as a standard (0·1 c.c. = 100), the Standard Malt Extract gave the remarkably high diastasic value of 143. As many of the malt extracts on the market have a much lower value than this, we are pleased to be able to recommend this preparation to our readers as one of genuine value. The same company's malt extract and cod liver oil is, however, capable of considerable improvement, as not only is its diastasic value much less, amounting in the sample examined to only 66 points, but the quantity of cod liver oil incorporated is very small, being only 5·61 per cent. The uncertainty of these mixtures of malt and cod liver oil make it very desirable that medical men and patients should not be misled in assuming that in taking these mixtures an ordinary dose of cod liver oil is being absorbed. The quantity present in the sample of the Standard Malt Extract Company's mixture is smaller than that found in other similar preparations, and the diastasic value being high, is in itself not sufficient recommendation for using it. We think it highly desirable that manufacturers selling malt and cod liver oil should state clearly on the bottle the amount of oil in percentage that is incorporated with the extract, and thus allow questions of price to decide the advantages of rival makes.

### "SANITAS" EUCALYPTUS SOAP.

We have received a specimen of the above from the Sanitas Company, Bethnal Green, London, for examination. Chemically, we have proved it to be what it claims to be, viz., a superfatted soap, containing oil of eucalyptus and the peculiar turpentiny principle, "Sanitas." It is a well-made article, and presents for antiseptic use a combination of volatile substances, all of which are known to be inimical to germ life. As it is practically impossible to prepare a metallic salt in combination with a soap basis without chemical reaction and consequent loss of germicidal power, so we are compelled, in our search for soapy antiseptics, to fall back on organic substances, which do not lose their power when mixed with soap, and this has been well gained in the combination we note above.

